# The current state of genetic counseling and newborn screening: an interview with Megan Tucker

**DOI:** 10.4155/fsoa-2017-0017

**Published:** 2017-03-20

**Authors:** Megan Tucker

**Affiliations:** 1 Indiana State University, Genetic Counseling Program, Terre Haute, IN, USA

**Keywords:** genetic counseling, genetics, genomics, newborn screening

## Abstract

**Megan Tucker talks to Francesca Lake, Managing Editor:** A certified genetic counselor for over 10 years, Megan is currently the director of the Indiana State University Genetic Counseling Graduate Program and the Genetic Counseling Clinic at Union Hospital (Terre Haute, IN, USA). She began her career split between the Center for Prenatal Diagnosis and the Medical Genetics and Neurodevelopmental Center at St Vincent Hospital (Indianapolis, IN, USA). During this time she was instrumental in both the development of the statewide Perinatal Loss Evaluation Program and a hospital protocol to ensure collection of cord blood to allow time to effectively genetically evaluate babies. Her current clinical focus is in cancer and psychiatric genetic counseling.

## Q Can you tell us a little about your background & what led you into genetic counseling?

My background is actually in education. My undergraduate bachelor's degree was in secondary education, and I taught high school biology and zoology and things like that. During my second year of teaching I realized that I loved the science content and the teaching, but I hated the disciplinary side of education. As a result I got online and searched for master's degrees in genetics, because that was just a particular interest that I had. I stumbled across genetic counseling, and it looked like a perfect fit for me. A lot of people seem to have gotten into the career that way, where they kind of stumbled into it – they did not go to their undergraduate universities thinking, “I am going to become a genetic counselor”. Genetic counselors are often people who heard about it in a class or stumbled on it after they graduated or something like that. I definitely fit that classic pattern. Nowadays, more people are hearing about it at younger ages and people are picking their undergraduate course work with an intent on a career of genetic counseling. That is my brief story and I have loved it ever since. It has been a good fit for me.

## Q What would you say is a typical day in the life of a genetic counselor?

A typical day is really going to vary based on where a genetic counselor works, and that is an exciting thing about the career – not everyone has the same day, same job or same specialty. Most genetic counselors, perhaps about 70% of them, actually see patients on a daily basis and probably work in a hospital/clinical setting [1]. That is what I did for the last 10 years prior to coming to Indiana State University (Terre Haute, IN, USA), to direct the training program and clinic. I saw between 2–5 patients on any given day with the average genetic counselor seeing approximately 10 patients per week [1]. Most consulations will take between 30 min to 1.5 h depending on whether it is a new patient and the reason for the referral. During that time the genetic counselor will gather a lot of information such as discussing the reason for the visit, the patient's goals, their medical history, their family history and we may work with their physicians to document a physical exam if necessary. Genetic counselors are often integral in viewing the plan with the family, and coordinating any aspects of care that are needed. We may discuss for example, is genetic testing warrented, what does that involve, what are we looking for, are there management changes or other evaluations that are needed, among other questions. Consultations could also be for a family that has already had genetic testing and we are focusing on the results and how it impacts the family. Again, the question may be, what do we do about it? What are the chances for other family members to be affected, and so on? We spend time making sure the families are adapting to the information by assessing their social and emotional support network, decision-making strategies and identifying useful resources and things like that.

For those that work in the clinic, an individual day may consist of actual patient visits and coordinating a variety of aspects of patient care. Even though I might only spend a few hours a day face-to-face with patients, I spend a lot of time coordinating the testing – reaching out to the insurance companies to see if that testing will be covered or writing letters to the patients or healthcare providers regarding the results and so on. This is in addition to other aspects of my day such as researching abnormal results or contacting patients by phone or email for follow-up which may include test results, answering questions, among others. Often, we might get a result that is not clear, so I have to spend some time reaching out to colleagues, reading primary literature to understand what this genetic change means, how we treat it, and so on. There are a lot of other apsects of my day too, like educating healthcare providers or the community on various topics related to genetics and healthcare, researching available clinical trials, or specialized clinics/protocols, and teaching or supervising students. For a counselor that works in a clinical-type setting, your day is often broken up between all these different things.

The other 30% of genetic counselors may not see patients on a daily basis. They may work in results interpretation in a laboratory, or in sales as a science medical liason, at an insurance company, academics, or in governmental positions dealing with public policy, among others. There are lots of different areas that counselors end up in.

## Q Has the role changed much over time?

It used to be that most genetic counselors graduated from a training program and worked in a hospital/clinical setting to see patients. It has been much more recent that there has been this shift where we are working in a lot of different areas, which is really exciting.

## Q What would you say are the most rewarding & indeed the most challenging aspects of your job?

What I enjoy the most and what is most challenging are a little bit one and the same. For me, finding an answer for the family is the most rewarding. I am curious; I want to know what is going on, so when I find an answer it is a great feeling. The families are usually very appreciative of that and so that is kind of the fun of it. But at the same time, sometimes the answer is not a good one; sometimes the answer is something that is devastating. As a result, even though the answers are the most rewarding, they can be the hardest situations when I have to sit down with the family and give them bad news or something they are not expecting. Sometimes the answer is something that we can do something about; we can treat it or at least give them information to better understand it and make plans for the future; and those instances are very rewarding.

## Q Why is the demand for genetic counselors outstripping supply in the USA?

I think a lot of it comes down to two things. One is the expansion of the role of the genetic counselor itself. Genetic counselors are going into these other areas – they are research coordinators, they are working in the lab and they are working in insurance, and so we have diversified our employment opportunities beyond clinical care. For example, some hospitals have begun using genetic counselors to evaluate the genetic tests that are ordered through their facility. These counselors may be looking for unnecessary or duplicated testing. Data has shown that by using genetic counselors in this way, companies can save money [2]. Even within the clinical setting, new specialties are starting to utilize genetic counselors. Historically, genetic counselors practiced in primarily prenatal settings, so clinics were seeing couples with babies at an increased risk for complications. That was really the bread and butter for genetic counselors. We also had people that worked in pediatrics, and then overtime the profession added cancer genetics, which has become one of the larger specialties for genetic counselors [1]. In addition to these three areas, we have also started to add cardiology, specialty clinics such as metabolism, connective tissue disorders, neurology, pharmacogenetics, among others. Thus, not only are we expanding outside of the clinic, we are also expanding the role of genetic counselors within the clinic, and so the whole field is growing rapidly.

However, there is such a heavy face-to-face training component that the genetic counseling programs have a hard time adding a lot of students. As a result, even though the job market is expanding, the genetic counseling training programs may only be adding a few students a year. The NSGC (National Society of Genetic Counselors) Professional Status Survey, has estimated that the growth rate from 2014 to 2024 genetic counselors will be approximately 29% compared to 7% for all occupations [1]. Even if one to three training programs are added annually, they each only add four to ten students to the total graduating genetic counselors. Therefore, the pool of genetic counselors is growing but it is not growing at the same rate as the need.

## Q What would you suggest we need to do to meet demand?

As directors we meet frequently as a group to discuss this, and the most obvious answer for most of us is changing our expectation of the face-to-face clinical experience that students get. Right now in most graduate programs in the USA and Canada, a portion of their 2-year training is spent in a clinical setting seeing patients. A critical part of becoming a genetic counselor is to get first-hand experience of what it is like to talk with a patient. Each student generally sees maybe 150, some of them up to 300 patients by the time they graduate. Finding that patient volume, when a program has ten students and each student is trying to see up to 150–300 patients means thousands of patients have to be identified for each student cohort. As such, to increase even by one or two students, you need to increase by hundreds of available patients. The question is therefore how do we change that – how do we gain the same skills that you are gaining by sitting down one-on-one with a real patient without necessarily sitting down with an actual patient? Some programs are doing things like patient simulations where actors come in and play the part of a patient. Thus, students are still gaining those first-hand skills but we are not taking away from the actual patient population that is out there. There is also a lot of movement to problem-based learning and online opportunities where students walk through scenarios or answer questions about what their decision-making would be if a patient said certain things. So, our goal right now is to try to use more of these alternative training methods so that we can expand our number of students without burdening the clinics and the supervising counselors. That is a big shift that we are all focusing on how to make.

## Q A recent investigation by Samantha Zent into newborn screening in the USA highlighted the controversies that exist over who owns genetic information [3]. What are your personal thoughts on this?

A lot of the controversy that has come up in the last few years about this comes from a lack of informed consent. Newborn screening was almost an assumed test. All that was said to me when my baby was born was, “we're going to do this heel stick and it's going to look for things we want to know about your baby's health”, and now all of a sudden there is all this genetic data around. Families were not necessarily being informed that these data were just sitting there and someone had the potential to be doing research on it. I think part of the controversy came from people's sudden realization that their personal information was sitting in a laboratory storage facility and they did not even know that. I think if we were able to sit down and explain this to patients this would be less of an issue, and there is definitely a shift to doing it more. Having a real informed consent process where the patient actually understands what is happening and who is going to have access to these data before they just automatically get tested is a really important key component, so that everybody is upfront on how the process works. That is certainly improving, and now that there have been controversies, people have realized that we need to make sure everybody is on board with what is happening so that the families get some options to say, “yes, it's okay for you to do further research on this” or “no, it's not, you need to destroy my sample”. They should have the right to say those kinds of things and make a decision about what happens with their DNA.

## Q It has also been suggested there is an issue with a lack of understanding in the general population about newborn screening. Is that still an ongoing issue?

There is still very little true discussion with families prior to testing. At the same time I do not know what the right answer to this problem is because it is not like we have enough genetic counselors to sit down with every woman that delivers a baby to be able to say, “here's the full information; what we're looking for, what it means and the risks and the benefits, and so on”. As a result, it is a hard place to be because there are not enough of us that know and understand the testing and so we have to rely on other medical professionals who are capable of doing it but who may not have the time or truly have the understanding. Also, patients might not even want to discuss it; they have just had a baby, they do not want to sit down and talk about these things. I do not have a perfect answer necessarily but this is still something that is a challenge because it is rare that you find something that is abnormal so families just assume that, “yeah, I'll do it, because it's not going to identify anything” and then the panic comes when something is found. All of a sudden this family is thrown into this spiral of medical visits and further testing and they were not expecting that. I do not think it is fair if they do not know what to expect but it is a hard hole to fill because there are not enough people to sit down with these individuals and fill that hole entirely.

## Q Does this misunderstanding affect your work?

Yes, it can because by the time they have gotten to me they have already had a newborn screen which was abnormal. Someone has called them and said, “there is this abnormal finding, you so need to go see a genetic counselor”. So, by the time they come to me, some of that initial confusion is still there but I am not the one breaking the news. As a result, when they sit down with a genetic counselor, that is our opportunity to say, “let's start from the beginning, here's the test you had, here's what it looked for, here's why we did it, and what it means for you”. While it does mean that I need to do a little bit more background discussion, that is not outside of my normal day anyway. We tend to get people who do not feel like they came in with an understanding of what was happening. Whereas, the people that get referred to us for a standard genetic evaluation, where we have the full discussion followed by genetic testing, typically do not have that same level of confusion when the results come back because they were prepped for what to anticipate. The patients that are getting abnormal newborn screens are lacking the preparation. I can handle that, but it increases frustration sometimes. It is important to realize that newborn screening is unique. Most patients that are picked up have treatment options; things that we can change to improve the outcome for those babies. Those families are generally very eager to learn more, to know what to expect and are usually very appreciative of the information. Unfortunately, there is often some confusion getting in the door that needs clarified.

## Q What other issues do genetic counselors face?

In relation to newborn screening I think sometimes the other piece of confusion is a normal result. Sometimes families come in and say, “well I did that newborn screening and it was normal, why is my child having developmental delays?” A newborn screen is designed to identify families that may benefit from further testing. It is not considered diagnostic nor can it completely rule out all conditions for which it screens. In addition, each state is different in the number of disorders considered part of their newborn screen. Families do not always understand what a test is evaluating and think that the newborn screen is testing for everything possible, which it is not. This can lead to frustration especially if the testing is not done with comprehensive informed consent. A normal result cannot rule out everything possible and in fact is only evaluating a small number of conditions.

## Q Finally, what would you say the field of genetic counseling will look like in 10–20 years’ time?

It has changed so much in the last 10–20 years I can only envision it continuing to expand and change. I think the biggest shift is probably going to be a part of the big shift in healthcare as a whole. This is toward the direction of personalized medicine and genomic testing. Right now, we do not have a lot of reliable means of doing genetic testing to assess someone's ultimate risk for common medical conditions (heart disease, diabetes and high cholesterol, among others) but I think that this is the direction that things are really moving in. Not only is it toward personalized medicine, it is toward prevention – for example, you might get a genetic screen that says you have a higher risk for developing Type II diabetes, and so you are going to modify your diet even before you develop Type II diabetes to try to prevent it. I think there is a lot of push in this area. There are also companies coming on board that are willing to take the bullet and develop medications and treatments for rare disorders, knowing that each one of them is incredibly unlikely to occur. But, if you lump rare disorders together, they count for a large percentage of hospitalizations. Developing a treatment for one of those and therefore improving outcomes for patients with these rare genetic disorders, may lead to additional treatments for others. I think both branches are growing quite rapidly. Studies have shown that current physicians do not feel confident in interpreting genetic testing results and those that are being trained, lack the same genetic knowledge as genetic counselors [4]. This means genetic counselors are in a unique position because of their science knowledge and ability to communicate complex information with families, identify resources and research novel situations. I think we are going to become more and more involved in these different areas by continuing to be at the forefront of using genetics in healthcare.
